# Supplementary material for: Proteome Map of Pea (Pisum sativum L.) Embryos Containing Different Amounts of Residual Chlorophylls
Source: Int J Mol Sci. 2018 Dec 15;19(12):4066. doi: 10.3390/ijms19124066 (PMC6320946; doi:10.3390/ijms19124066)
Supplement: Supplementary file 1 [file ijms-19-04066-s001.zip › Supplementary information/Mamontova_et_al_Supplementary_information_1.docx]

**Proteome map of pea (*Pisum sativum* L.) embryos containing different amounts of residual chlorophylls**

Tatiana Mamontova,^1,2§^ Elena Lukasheva,^2§^ Gregory Mavropolo-Stolyarenko,^2§^ Carsten Proksch,^3^ Tatiana Bilova,^1,4^ Ahyoung Kim,^1^ Vladimir Babakov,^5^ Tatiana Grischina,^2^ Wolfgang Höhenwarter,^3^ Sergei Medvedev,^4^ Galina Smolikova^4*^ and Andrej Frolov^1,2*^

**Supplementary information 1**

^1^Department of Bioorganic Chemistry, Leibniz Institute of Plant Biochemistry, ^2^Department of Biochemistry, St. Petersburg State University, ^3^Proteome Research Unit, Leibniz Institute of Plant Biochemistry, ^4^Department of Plant Physiology and Biochemistry, St. Petersburg State University and ^5^Research Institute of Hygiene, Occupational Pathology and Human Ecology

^§^These authors contributed equally to the manuscript

*Corresponding authors:

Dr. Andrej Frolov

Leibniz Institute of Plant Biochemistry

Department of Bioorganic Chemistry

Weinberg 3,

06120, Halle/Saale, Germany

Tel. +49 (0) 345 55821350

Fax. +49 (0) 345 55821309

Email: afrolov@ipb-halle.de

Dr. Galina Smolikova

St. Petersburg State University

Dep. of Plant Physiology and Biochemistry

Universitetskaya nab. 7-9,

199034, St. Petersburg, Russia

Tel. +7 812 3289695

Fax. +7 812 3289703

Email: g.smolikova@spbu.ru

Directory

[**Protocols** 3](#_Toc526851180)

[**Protocol S1-1** Determination of chlorophylls and carotenoids 3](#_Toc526851181)

[**Protocol S-2** Quantification of hydrogen peroxide 4](#_Toc526851182)

[**Protocol S-3** Determination of malondialdehyde (MDA) contents 5](#_Toc526851183)

[**Protocol S1-4** Sodium dodecyl sulfate-polyacrylamide gel electrophoresis (SDS-PAGE) 6](#_Toc526851184)

[**Protocol S1-5** Digestion of isolated total plant protein fraction with trypsin 7](#_Toc526851185)

[**Protocol S1-6** Analysis of protein networks in String database 8](#_Toc526851186)

[**Tables** 10](#_Toc526851187)

[**Table S1-1** Parameters of the nanoHPLC separation method 10](#_Toc526851188)

[**Table S1-2** Instrument settings applied for ESI-Orbitrap-Q-MS DDA experiments 11](#_Toc526851189)

[**Table S1-3** Sequence databases used for building a combined legume database 13](#_Toc526851190)

[**Table S1-4** Proteome Discoverer settings, used for Sequest database search 17](#_Toc526851191)

[**Table S1-5** Protein recoveries and total UV densities 18](#_Toc526851192)

[**Table S1-6** Depletion of pea seed high-abundant proteins with protamine sulfate 19](#_Toc526851193)

[**Figures** 20](#_Toc526851194)

[**Figure S1-1** Mature dry seeds with yellow and green cotyledons 20](#_Toc526851195)

[**Figure S1-2** Automatic data processing pipeline 21](#_Toc526851196)

[**Figure S1-3** SDS-PAGE of individual protein samples 22](#_Toc526851197)

[**Figure S1-4** SDS-PAGE of tryptic digests 23](#_Toc526851198)

[**Figure S1-5** SDS-PAGE of individual protein samples with and without depletion 24](#_Toc526851199)

[**Figure S1-6** SDS-PAGE of individual protein samples with and without depletion 25](#_Toc526851200)

[**Figure S1-7** SDS-PAGE of tryptic digests with and without depletion 26](#_Toc526851201)

[**Figure S1-8** Prediction of sub-cellular localization of proteins 27](#_Toc526851202)

[**Literature** 29](#_Toc526851203)

# Protocols

# Protocol S1-1 Determination of chlorophylls and carotenoids

Determination of pigments (n = 9) relied on the method of Lichtenthaler и A.R.Wellburn [1]. In detail, approximately 100 mg of embryos were homogenized in 500 µL of methanol. After centrifugation (1500 g, 10 min, room temperature), the supernatants were transferred in new tubes. The extraction was repeated four times to achieve the complete discoloration of tissues, before the total extract volume was adjusted to 2 mL with methanol. The contents of seed chlorophylls and carotenoids in methanolic extracts were determined spectrophotometrically, using a Implen NanoPhotometer P360 (Implen GmbH, Schatzbogen, Germany) at 470, 653, 666 and 700 nm (baseline).

Calculations:

C*_a_* = 15.65 × A_666_ – 7.34 × A_653_

C*_b_* = 27.05 × A_653_ – 11.21 × A_666_

C_x+c_= (1000 × A_470_ – 2.86 × C*_a_* – 129.2 × C*_b_*) / 221

where

C*_a_* – concentration of chlorophyll *a* (µg/ml)

C*_b_* - concentration of chlorophyll *b* (µg/ml)

C_x+c_ – concentration of xanthophylls (x) and carotenes (c, µg/ml)

A*_n_* – absorption at the wavelength *n* nm

# Protocol S-2 Quantification of hydrogen peroxide

Quantification of hydrogen peroxide relied on the method of Bilova *et al* [2]. In detail, approximately 100 mg of the plant material were extracted with 1 mL of ice-cold 0.4 mol/L perchloric acid. Samples were vortexed for 30 s and centrifuged (10 000 g, 10 min, 4 °C). The supernatant was neutralized with KOH, diluted four-fold with sodium phosphate buffer (0.1 mol, pH 5.6) and supplemented with ascorbate oxidase (8 units, 2 µL in 4 mmol/L sodium phosphate buffer pH 5.6, 10 min, RT). Afterwards, two aliquots (500 µL each) were transferred to new polypropylene tubes, with one of them treated with catalase (50 units in 2 µL in 4 mmol/L sodium phosphate buffer pH 5.6, 2 min, RT). Both aliquots were supplemented with an equal volume of the FOX reagent (0.2 mmol/L xylenol orange, 200 mmol/L sorbitol, 50 mmol/L H_2_SO_4_, and 0.5 mmol/L (NH_4_)_2_ Fe(SO_4_)_2_), and incubated for 30 min in the dark before measurement of the Fe(II)-xylenol orange complex absorption at 575 nm. The values obtained for the catalase-treated samples were subtracted from those of the catalase-free ones, to obtain the corrected optical densities. The calibration was performed externally by an H_2_O_2_ serial dilution series (1–10 μmol/L).

# Protocol S-3 Determination of malondialdehyde (MDA) contents

The MDA contents were determined as equivalents of thiobarbituric acid (TBA) using the method of Frolov and co-workers [3]. In detail, approximately 25 mg of frozen grinded plant material were left on ice for 3 minutes, before addition of 300 µL 5% (w/v) trichloroacetic acid (TCA), vortexed for 30 s and centrifuged at 10000 g for 20 minutes at 4°C. 250 µL of supernatant were transferred in a new polypropylene tube, and 1000 µL of thiobarbituric acid (TBA) reagent (0.5 % w/v TBA in 20% TCA) were added. The mixture was incubated for 30 min in boiling water bath (95°C). Afterwards, the mixture was cooled on ice to stop the reaction, centrifuged at 1900 g for 10 minutes at 4°C and 1 ml of colored supernatant was used to measure the absorbance at 532 nm against the proper blank (250 µL 5% w/v TCA and 750 µL TBA reagent). The non-specific absorbance at 600 nm was subtracted from the absorbance acquired at 532 nm. The contents of MDA equivalents were calculated with ε = 155 mM^-1^cm^-1^

# Protocol S1-4 Sodium dodecyl sulfate-polyacrylamide gel electrophoresis (SDS-PAGE)

SDS-PAGE was done with a 12% resolving and a 6% stacking (gel T=12%, C=2.65%). Typically, aliquots of protein samples, corresponding to 10 µg, were completely dried under reduced pressure and reconstituted in 20 µL of sample buffer, containing 0.05% (w/v) bromophenol blue, 20% (v/v) glycerol, 2% (w/v) SDS, 5% (v/v) β-mercaptoethanol in 62.5 mmol/L Tris-HCl (pH 6.8). On each lane, 10 µL of this solution (corresponding to 5 µg of protein) were loaded. Aliquots of tryptic digests, corresponding to 5 µg, were completely dried under reduced pressure and reconstituted in 10 µL of the same buffer. The whole volume of the solution was loaded on each lane in this case. The molecular weights of proteins, representing individual electrophoretic zones were determined by a molecular weight standard mix, run on the same gel. After completion of separation (45 min at 200 V), gels were stained with Coomassie Brilliant Blue G-250 for 1 h. Average densities across individual lanes (expressed in arbitrary units) were determined by ChemiDoc XRS imaging system controlled by Quantity One^®^ 1-D analysis software (Bio-Rad Laboratories Ltd., Moscow, Russia). For calculation of relative standard deviations (RSDs), the densities of individual lines were normalized to the gel average value.

# Protocol S1-5 Digestion of isolated total plant protein fraction with trypsin

The digestion procedure relied on the protocol of Frolov and co-workers [3] with minor modifications. In detail, aliquots of protein (32 or 50 µg of depleted and non-depleted fractions, respectively) were supplemented with 10 µL of 50 mmol/L TCEP in AALS-free shotgun buffer and diluted to obtain a total volume of 100 µL. After a 30-min incubation at 37 °C under continuous shaking (450 rpm), and cooling the samples to room temperature (RT), 11 µL of 0.1 mol/L iodoacetamide in shotgun buffer were added, and the mixture was incubated for 60 min at 4 °C in darkness. Afterwards, 875 µL of 50 mmol/L ammonium bicarbonate were pipetted to each sample, and trypsin (0.5 g/L in the same solution) was sequentially added twice at the enzyme-protein ratio of 1:20 and 1:50. Proteolysis was performed at 37°C under continuous shaking (450 rpm) for 5 and 12 h, respectively. The completeness of tryptic digestion was confirmed by SDS-PAGE (as described above), AALS was destroyed by addition of 111 µL of 10% (v/v) trifluoroacetic acid (TFA, final concentration 1% v/v) and incubation for 20 min at 37°C under continuous shaking (450 rpm). After this, the digests were desalted by solid phase extraction (SPE) using in-house prepared stage-tips, i.e. polypropylene pipette tips (200 µL) filled with six layers of C18 reversed phase material (Empower™ SPE discs). The eluents were driven by centrifugal force (1500 g) after placing stage-tips in 2 mL polypropylene tubes using appropriate adaptors. The stage-tips were conditioned with 100 µL of methanol, equilibrated with two portions (200 µL) of 0.1% (v/v) TFA, before samples were loaded and washed with two 200 µL-portions of 0.1% (v/v) formic aid (FA). Afterwards, stage-tips with adaptors were transferred in new polypropylene tubes and retained peptides were sequentially eluted with 40, 60, and 80% (v/v) acetonitrile in aqueous (aq.) 0.1% (v/v) FA, as proposed by Spiller and co-workers [4]. The resulting eluates were freeze-dried overnight under reduced pressure in a CentriVap Vacuum Concentrator (Labconco, Kansas City, USA) and stored at -20 °C before analysis.

# Protocol S1-6 Analysis of protein networks in String database

For analysis of protein networks in String database, accession numbers of the Universal Protein Resource Knowledgebase (UniprotKB) or Kyoto Encyclopedia of Genes and Genomes (KEGG) of 251 pea proteins, unique for green seeds (Figure 8A and Supplementary information 6) were submitted in the corresponding databases to retrieve the protein sequences in FASTA format. The obtained data for all proteins were combined in one FASTA file (Supplementary information 7), which then was searched for the protein homology against the *Arabidopsis thaliana* proteome in String database (<https://string-db.org/>)[5]. The analysis revealed 237 proteins, for which *A. thaliana* homologs could be found. Thereby, each query protein was matched to several *A. thaliana* protein homologs, ranked in the identity and bit score descending order. Finally, only the *A. thaliana*proteins with the highest bit score for corresponding query proteins were selected for the network analysis (Supplementary information 7).

At the next step,to extract only highly confident protein-protein interaction networks, the following filters were applied:

| **Parameter** | **Setting** |
| --- | --- |
| Score | ≥0.7 (high and very high confident interactions) |
| Max. number of interactions to show | 1^st^ shell (query proteins only) |
| Disconnected nodes in the network | Hidden |
| Type of interactions | Experiment*^a^*  Co-expression*^b^* |

The interactions relied on experimentally derived functional and/or structural evidences *^a^*and*^b^*co-expression data

The nodes of the networks denote interacting proteins, whereas the lines connecting two neighboring nodes represent protein-protein interactions. The node colors have no biological meaning, whereas the color of their filling indicates the presence of the protein structural information. The line colors denote the types of interaction.

At the last step, the functional pathway information of proteins from each claster was manually retrieved and analyzed in work with the databases UniprotKB, KEGG, TAIR (Arabidopsis Information Resource), ARAPORT (Arabidopsis Informational Portal, <https://www.araport.org/>, [6]). Detailed description of the protein data extracted from the protein networks is provided in Supplementary information 7.

# Tables

# Table S1-1 Parameters of the nanoHPLC separation method

| **Parameter** | **Setting** | |
| --- | --- | --- |
| Injection volume  Sample pickup flow | 1 µL  20 µL/min |  |
| Injection mode | Full loop injection at 800 bar |  |
| Eluents | A: 0.1% (v/v) aq. formic acid; B: 0.1% (v/v) formic acid in acetonitrile |  |
| Elution flow rate | 0.3 µL/min |  |
| Column temperature | 40⁰C |  |
| Elution regimen | Linear gradient - from 5 to 36% eluent B in 160 min  Linear gradient – from 36 to 38% eluent B in 50 min  Linear gradient – from 38 to 80% eluent B in 2 min  Isocratic at 80% eluent B during 8 min |  |
| Isocratic flow | Isocratic 75% eluent B till the start of the next run |  |
| Trap column equilibration | 12 µL of A at 800 bar |  |
| Analytical column equilibration | 4 µL of A at 800 bar |  |

# Table S1-2 Instrument settings applied for ESI-Orbitrap-Q-MS DDA experiments

| **Parameter** | **Setting** |
| --- | --- |
| **MS conditions** | |
| Ionization mode | Positive |
| Resolution | 70,000 |
| Ion spray voltage (IS) | 1900 V |
| Capillary temperature | 275 °C |
| Default charge state | 2 |
| Microscans | 1 |
| AGC Target | 3x10^-6^ |
| Maximum IT | 100 ms |
| Number of scan ranges | 1 |
| Mass to charge ratio (*m/z*) range | 400 – 1850 |
| Spectrum data type | Profile |
| **MS/MS conditions** | |
| Fragmentation | Collision activated dissociation |
| Microscans | 1 |
| Resolution | 17,500 |
| AGC target | 5x10^-4^ |
| Maximum IT | 50 ms |
| Loop count | 10 |
| MSX count | 1 |
| TopN | 10 |
| Isolation window | 1.6 *m/z* |
| Isolation offset | 0 m/z |
| Scan range | Automatic |
| Fixed first mass | 120 *m/z* |
| (N)CE | 28 |
| Spectrum data type | Centroid |
| Minimum AGC target | 1.5x10^-3^ |
| Intensity threshold | 3x10^-4^ |
| Charge state rejected | Unassigned, +1 < n < +7 |
| Peptide match | Preferred |
| Exclude isotopes | On |
| Duration of dynamic exclusion duration | 40 s |
| If idle | Do not pick others |

# Table S1-3 Sequence databases used for building a combined legume database

| **Full proteomes** | | | |
| --- | --- | --- | --- |
| **Nr** | **Species** | | **Sequences** |
| 1 | *Medicago truncatula* Gaertn | | From Uniprot,01.06.2017  From MTGD ProteinSeq v4.2, 18.08.2014 |
|  |  |  |  |
| 2 | *Lotus japonicas* (Regel) K.Larsen | | From Uniprot, 01.06.2017  From Lotus base, 01.06.2017  From Lotus base, 08.07.2017 |
| 3 | *Phaseolus vulgaris* L. | | From Uniprot, 16.03.2017 |
| **Sequences of the proteins, related to seed longevity** | | | |
| **Accession** | | **Source species** | **Protein name** |
| AJ498523 | | *Robinia pseudoacacia* | Em protein |
| O22850 | | *Arabidopsis thaliana* | Probable glutathione peroxidase 3, mitochondrial |
| Q9SL15 | | *Arabidopsis thaliana* | Glycine-rich protein 3 |
| Q03250 | | *Arabidopsis thaliana* | Glycine-rich RNA-binding protein 7 |
| Q9SVM8 | | *Arabidopsis thaliana* | Glycine-rich RNA-binding protein 2, mitochondrial |
| Q9LY09 | | *Arabidopsis thaliana* | Oleosin GRP-17 |
| Q9LIS2 | | *Arabidopsis thaliana* | Glycine-rich RNA-binding protein 4, mitochondrial |
| Q9LTP5 | | *Arabidopsis thaliana* | Glycine-rich protein 5 |
| O48848 | | *Arabidopsis thaliana* | Glycine-rich protein 23 |
| O48646 | | *Arabidopsis thaliana* | Probable phospholipid hydroperoxide glutathione peroxidase 6, mitochondrial |
| Q9LYB4 | | *Arabidopsis thaliana* | Probable glutathione peroxidase 5 |
| O04922 | | *Arabidopsis thaliana* | Probable glutathione peroxidase 2 |
| P52032 | | *Arabidopsis thaliana* | Phospholipid hydroperoxide glutathione peroxidase 1, chloroplastic |
| Q8LBU2 | | *Arabidopsis thaliana* | Probable glutathione peroxidase 8 |
| Q9SZ54 | | *Arabidopsis thaliana* | Putative glutathione peroxidase 7, chloroplastic |
| Q8L910 | | *Arabidopsis thaliana* | Probable glutathione peroxidase 4 |
| Q03251 | | *Arabidopsis thaliana* | Glycine-rich RNA-binding protein 8 |
| Q03250 | | *Arabidopsis thaliana* | Glycine-rich RNA-binding protein 7 |
| Q9SVM8 | | *Arabidopsis thaliana* | Glycine-rich RNA-binding protein 2, mitochondrial |
| Q9LIS2 | | *Arabidopsis thaliana* | Glycine-rich RNA-binding protein 4, mitochondrial |
| P0CJ48 | | *Arabidopsis thaliana* | Chlorophyll a-b binding protein 2, chloroplastic |
| Q9S7J7 | | *Arabidopsis thaliana* | Chlorophyll a-b binding protein 2.2, chloroplastic |
| Q9S7M0 | | *Arabidopsis thaliana* | Chlorophyll a-b binding protein 3, chloroplastic |
| Q9S7W1 | | *Arabidopsis thaliana* | Chlorophyll a-b binding protein CP29.3, chloroplastic |
| Q9XF89 | | *Arabidopsis thaliana* | Chlorophyll a-b binding protein CP26, chloroplastic |
| Q9LMQ2 | | *Arabidopsis thaliana* | Chlorophyll a-b binding protein, chloroplastic |
| Q9XFH8 | | *Arabidopsis thaliana* | Thioredoxin F1, chloroplastic |
| Q9C9Y6 | | *Arabidopsis thaliana* | Thioredoxin H9 |
| Q42403 | | *Arabidopsis thaliana* | Thioredoxin H3 |
| Q39241 | | *Arabidopsis thaliana* | Thioredoxin H5 |
| Q9SEU6 | | *Arabidopsis thaliana* | Thioredoxin M4, chloroplastic |
| O48737 | | *Arabidopsis thaliana* | Thioredoxin M1, chloroplastic |
| Q9SEU7 | | *Arabidopsis thaliana* | Thioredoxin M3, chloroplastic |
| P29448 | | *Arabidopsis thaliana* | Thioredoxin H1 |
| Q9SEU8 | | *Arabidopsis thaliana* | Thioredoxin M2, chloroplastic |
| Q9XFH9 | | *Arabidopsis thaliana* | Thioredoxin F2, chloroplastic |
| Q38879 | | *Arabidopsis thaliana* | Thioredoxin H2 |
| Medtr2g017540.1 | | *Medicago truncatula* | LEAm |
| Medtr4g109500.1 | | *Medicago truncatula* | SBP65 |
| G7IFN8 | | *Medicago truncatula* | Methionine sulfoxide reductase B |
| I3SN78 | | *Medicago truncatula* | Methionine sulfoxide reductase B 2 |
| G7LCY5 | | *Medicago truncatula* | Peptide methionine sulfoxide reductase family protein |
| A0A072TSD3 | | *Medicago truncatula* | Peptide methionine sulfoxide reductase family protein |
| G7IFP1 | | *Medicago truncatula* | Methionine sulfoxide reductase B 2 |
| G7JU02 | | *Medicago truncatula* | Methionine sulfoxide reductase B 2 |
| G7IFP3 | | *Medicago truncatula* | Methionine sulfoxide reductase B 2 |
| G8A2F8 | | *Medicago truncatula* | Peptide methionine sulfoxide reductase family protein |
| G7IXM5 | | *Medicago truncatula* | Peptide methionine sulfoxide reductase family protein |
| B7FJB1 | | *Medicago truncatula* | Peptide methionine sulfoxide reductase family protein |
| A0A072UW64 | | *Medicago truncatula* | Peptide methionine sulfoxide reductase family protein |
| A0A072UWN9 | | *Medicago truncatula* | Peptide methionine sulfoxide reductase family protein |
| A0A072UFH0 | | *Medicago truncatula* | Peptide methionine sulfoxide reductase family protein |
| G7K8J3 | | *Medicago truncatula* | Peptide methionine sulfoxide reductase family protein |
| O24648 | | *Pisum sativum* | Gibberellin 3-beta-dioxygenase 1 |
| O81822 | | *Arabidopsis thaliana* | 17.7 kDa class II heat shock protein |
| P09918 | | *Pisum sativum* | Seed linoleate 9S-lipoxygenase-3 |
| P14856 | | *Pisum sativum* | Seed linoleate 9S-lipoxygenase-2 |
| P19036 | | *Arabidopsis thaliana* | 17.4 kDa class I heat shock protein |
| P29830 | | *Arabidopsis thaliana* | 17.6 kDa class II heat shock protein |
| P42730 | | *Arabidopsis thaliana* | Chaperone protein ClpB1 |
| Q01593 | | *Arabidopsis thaliana* | B3 domain-containing transcription factor ABI3 |
| Q02973 | | *Arabidopsis thaliana* | Em-like protein GEA6 |
| Q42539 | | *Arabidopsis thaliana* | Protein-L-isoaspartate O-methyltransferase 1 |
| Q8LDU4 | | *Arabidopsis thaliana* | Red chlorophyll catabolite reductase, chloroplastic |
| Q8LP17 | | *Pisum sativum* | Carotenoid 9,10(9',10')-cleavage dioxygenase 1 |
| Q93ZA0 | | *Arabidopsis thaliana* | Probable chlorophyll(ide) b reductase NYC1, chloroplastic |
| Q9C5B9 | | *Arabidopsis thaliana* | Probable aldo-keto reductase 1 |
| Q9FFZ1 | | *Arabidopsis thaliana* | Pheophytinase, chloroplastic |
| Q9FYC2 | | *Arabidopsis thaliana* | Pheophorbide a oxygenase, chloroplastic |
| Q9LMQ2 | | *Arabidopsis thaliana* | Chlorophyll a-b binding protein, chloroplastic |
| Q9LVW2 | | *Arabidopsis thaliana* | Heat stress transcription factor A-9 |
| Q9XIE3 | | *Arabidopsis thaliana* | 17.6 kDa class I heat shock protein 1 |

# Table S1-4 Proteome Discoverer settings, used for Sequest database search

| **Database search settings** | |
| --- | --- |
| Analysis program | SEQUEST |
| Protease | Trypsin |
| Missed cleavage sites | 2 |
| MS (peptide) tolerance | 10 ppm |
| MS/MS tolerance | 0.02 *m/z* |
| Target FDR (strict) | 0.01 |
| Target FDR (relaxed) | 0.05 |
| Validation based on | *q* value |
| **Modification** | **Mass increment (Da)/amino acids** |
| Carbamidomethyl | 57.021/C^a^ |
| Oxidation | 15.995/M/W |

**^a^**Static modification

# Table S1-5 Protein recoveries and total UV densities calculated for individual samples separated by SDS-PAGE

| **Sample** | **Sample weight (mg)** | **Protein concentration**  **(mg/mL)** | **Protein recovery**  **(mg/g fresh weight)** | **UV densities (AU)^a^** |
| --- | --- | --- | --- | --- |
| YS-1 | 48.8 | 19.3 | 39.6 | 15400 |
| YS-2 | 48.7 | 20.7 | 42.5 | 14200 |
| YS-3 | 52.3 | 23.5 | 44.9 | 14500 |
| GS-1 | 48.8 | 33.5 | 68.7 | 15300 |
| GS-2 | 49.4 | 31.7 | 64.2 | 14900 |
| GS-3 | 49.9 | 61.9 | 124.1 | 14300 |

YS and GS denote the seeds of yellow- and green-seeded cultivars Millennium and Gloriosa, respectively; AU, arbitrary units;

**^a^**optical density was recodered at 595 nm

# Table S1-6 Depletion of high-abundant proteins in pea seed embryos with protamine sulfate

| **Sample** | **Protein concentration (mg/mL)** | |  | **Protein recovery (mg/g fresh weight)** | | **Efficiency of depletion (%)** |
| --- | --- | --- | --- | --- | --- | --- |
|  | **before depletion** | **after depletion** |  | **before depletion** | **after depletion** |  |
| YS-1 | 98,281 | 4,533 |  | 81,901 | 3,777 | 95,4 |
| YS-2 | 85,563 | 3,439 |  | 71,302 | 2,866 | 96,0 |
| YS-3 | 100,828 | 4,013 |  | 84,023 | 3,344 | 96,0 |
| GS-1 | 68,594 | 4,813 |  | 57,161 | 4,010 | 93,0 |
| GS-2 | 67,375 | 5,017 |  | 56,146 | 4,181 | 92,6 |
| GS-3 | 73,141 | 4,403 |  | 60,951 | 3,669 | 94,0 |

The major embryo proteins were depleted with 0.07% (w/v) protamine sulfate in 20 mmol/L MgCl_2_ in 0.5 mol/L Tris-HCl (pH 8.3) according the slightly modified procedure of Kim et al [7]. YS and GS denote the embryos of yellow and green seeds

# Figures


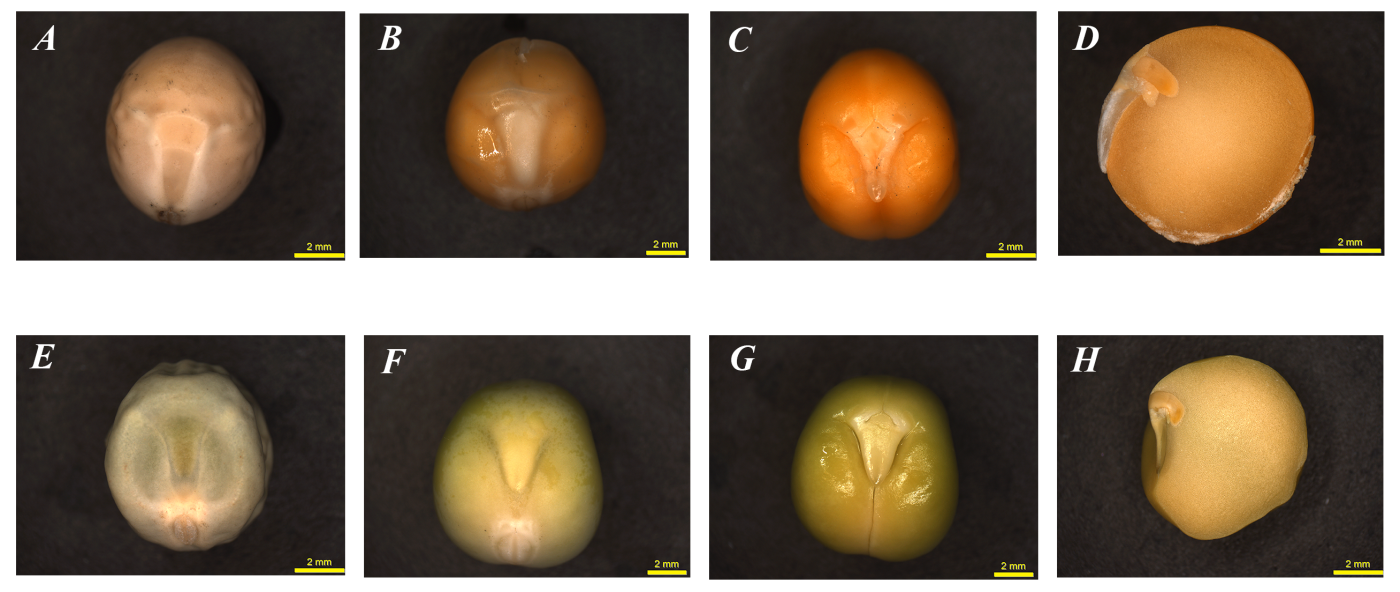


Figure S1-1 Mature seeds with yellow cotyledons (cultivar Millennium, A - D) and with green cotyledons (cultivar Gloriosa, E - H). A and E – dry seeds; B and F – seeds, imbibed for 1 h; C and G – embryos; D and H – embryos, longitudinal slice

*A*


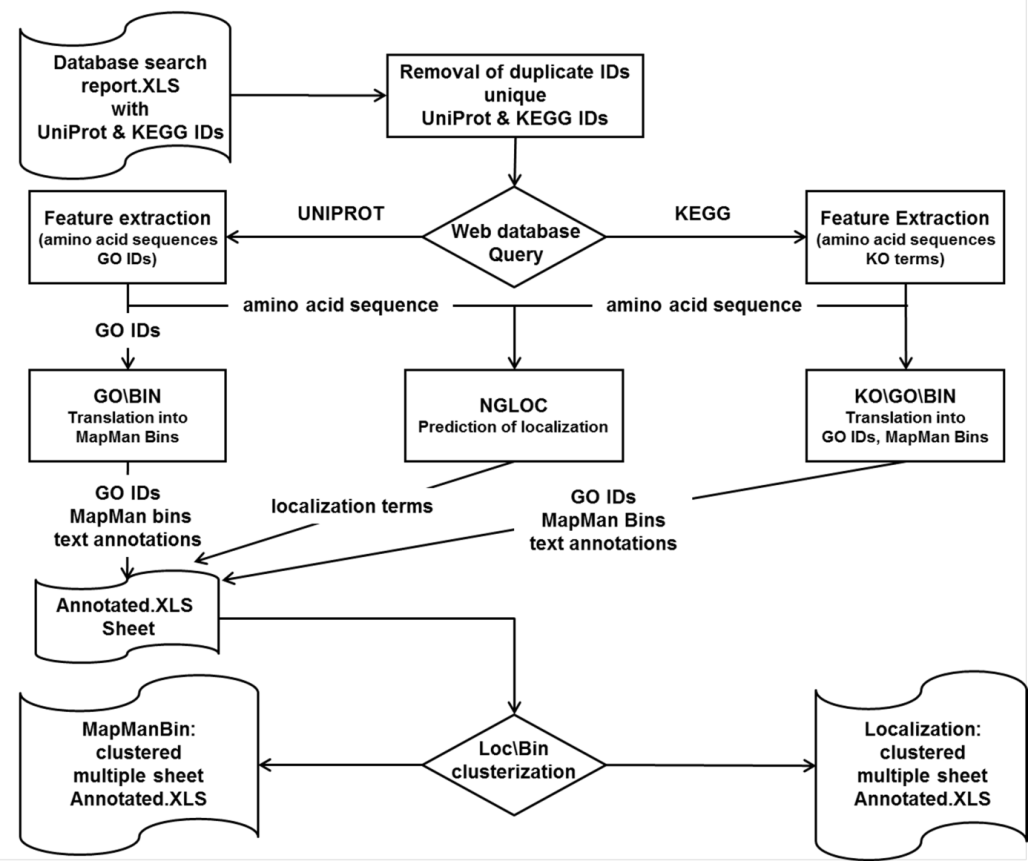


*B*

Figure S1-2 Automatic data processing pipeline for functional annotation of pea seed embryo proteins and prediction of their intra-cellular localization (A) and architecture of the data processing pipeline. Simple processing stages and decision\data split stages are denoted by rectangles and diamonds, respectively. XLS documents – sheets in Excel file.

**
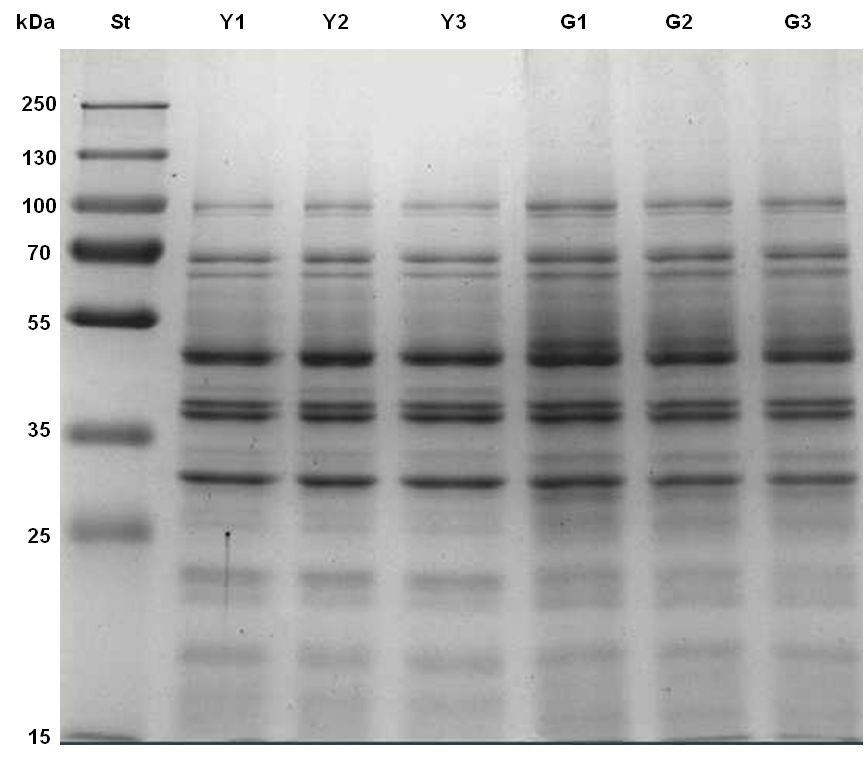
**

Figure S1-3 SDS-PAGE electropherogram of individual protein samples (5 µg) isolated from yellow (cultivar Millennium, Y1, Y2 and Y3) and green (cultivar Gloriosa, G1, G2, and G3) pea seed embryos; St, mixture of molecular weight standards


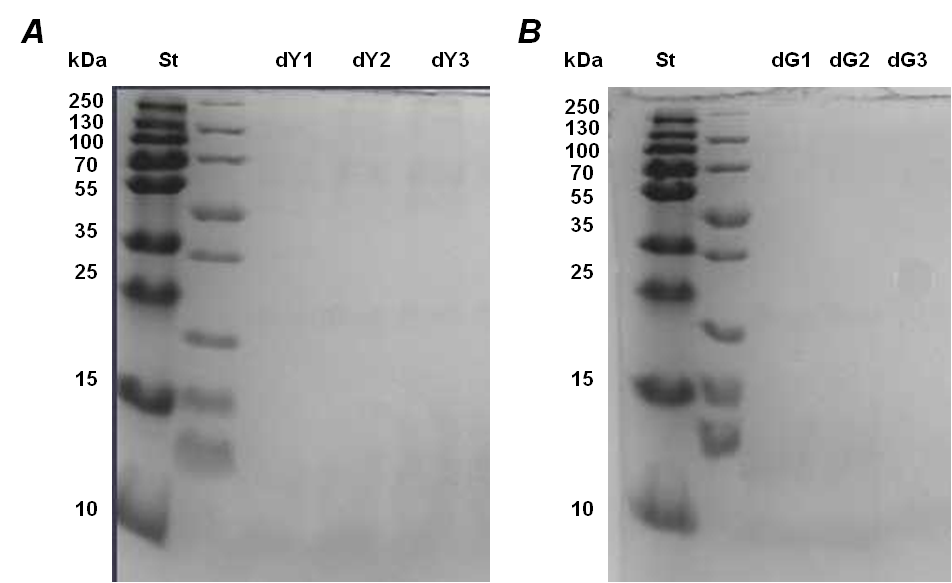


Figure S1-4 SDS-PAGE electropherogram of tryptic digests (5 µg) of the total protein fraction isolated from yellow (cultivar Millennium, d-Y1, d-Y2 and d-Y3, A) and green (cultivar Gloriosa, d-G1, d-G2, d-G3, B) pea seed embryos; St, mixture of molecular weight standards


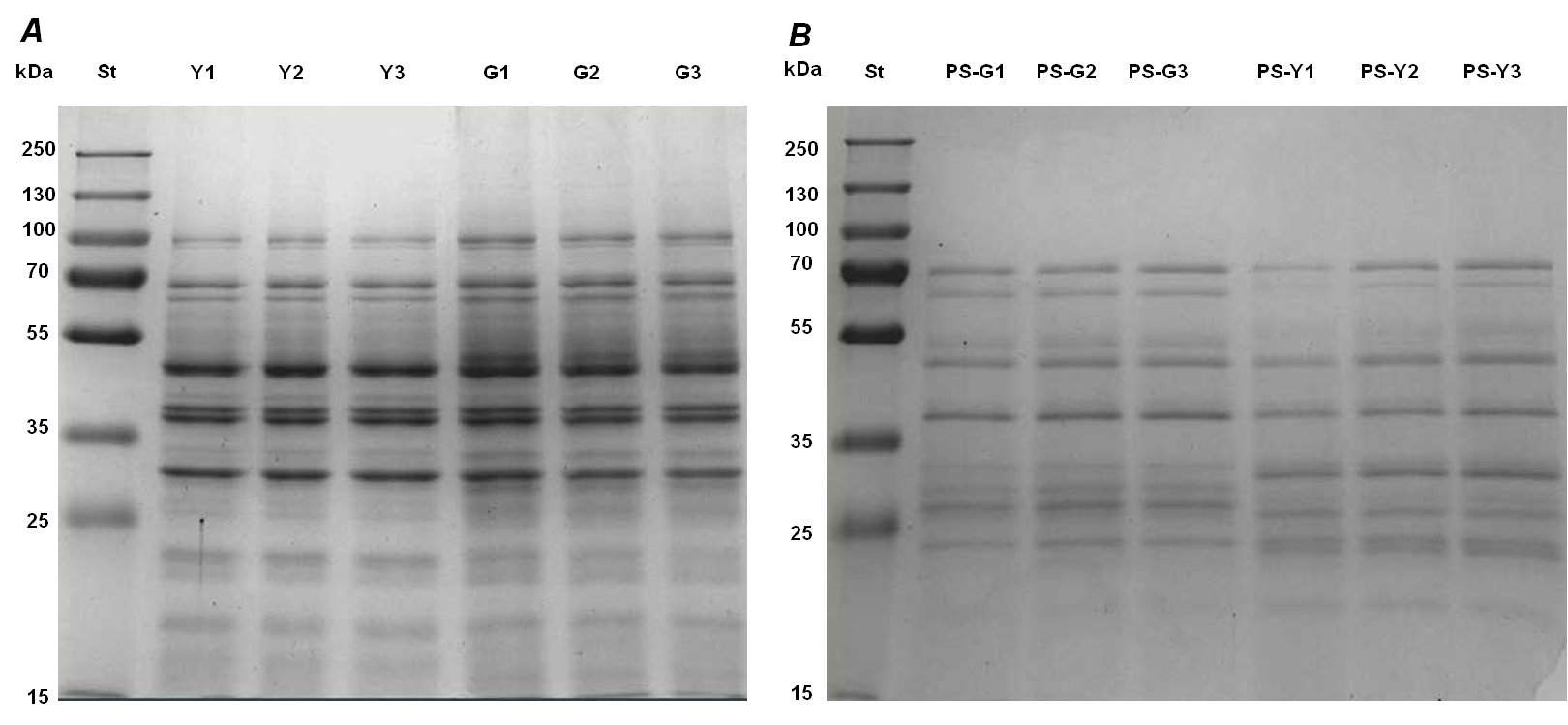


Figure S1-5 SDS-PAGE electropherogram of individual protein samples (5 µg) isolated from yellow (cultivar Millennium) and green (cultivar Gloriosa) pea seed embryos without (A) and with (B) depletion of highly-abundant proteins with 0.07% (w/v) protamine sulphate; St, mixture of molecular weight standards; Y1, Y2, Y3 and G1, G2, G3 denote the protein samples obtained from yellow and green seeds without depletion of highly-abundant proteins with protamine sulfate, respectively; PS-Y1, PS-Y2, PS-Y3 and PS-G1, PS-G2, PS-G3 denote the protein samples obtained from yellow and green seed embryos after depletion of highly-abundant proteins with protamine sulfate, respectively;

***
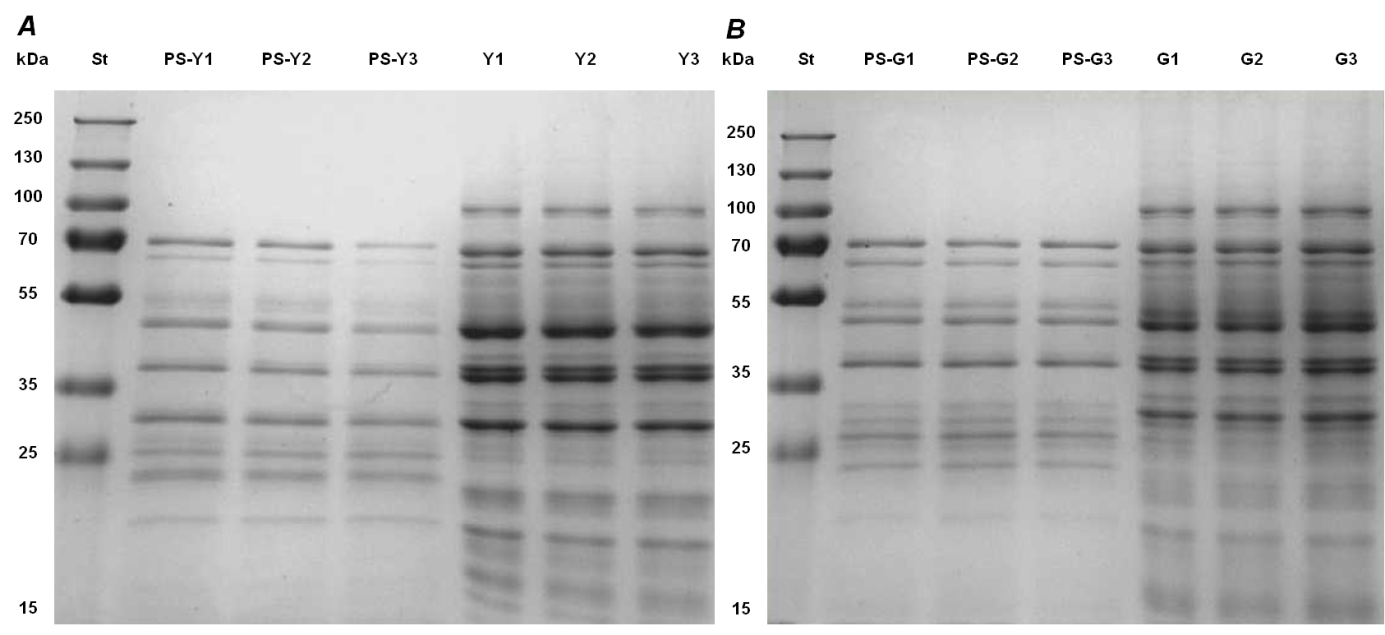
***

Figure S1-6 SDS-PAGE electropherogram of individual protein samples (5 µg) isolated from yellow (cultivar Millennium, Y1, Y2 and Y3, A) and green (cultivar Gloriosa, G1, G2 and G3, B) pea seed embryos with (labelled with prefix PS-) and without (without prefix PS-) depletion with 0.07% (w/v) protamine sulfate; St, mixture of molecular weight standards

**
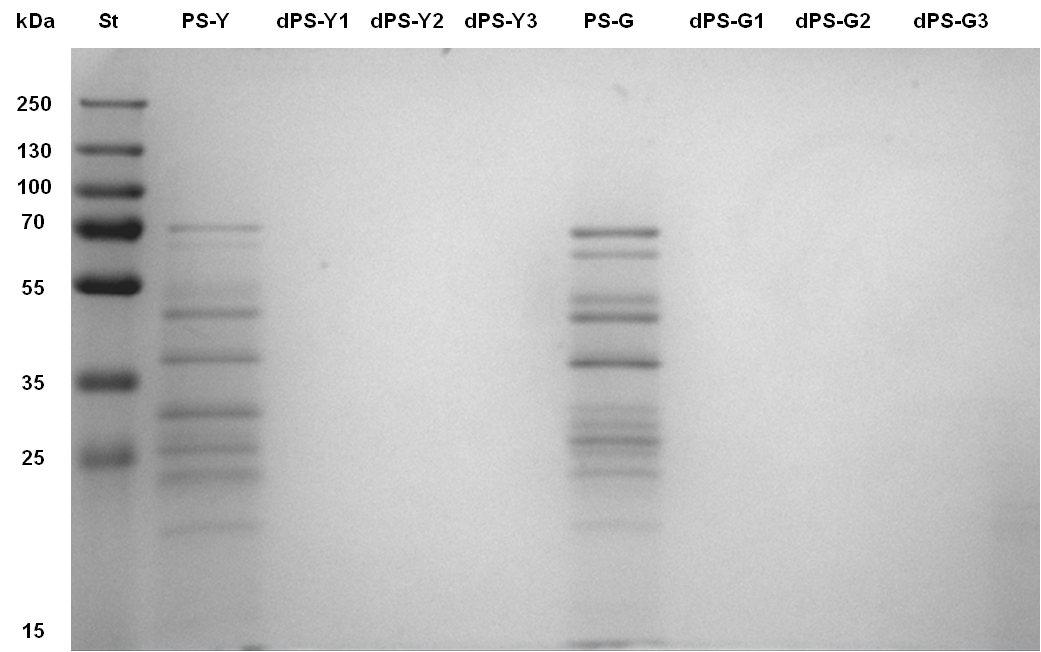
**

Figure S1-7 SDS-PAGE electropherogram of tryptic digests (5 µg loaded per line), obtained from protein fraction of yellow (cultivar Millennium, dPS-Y1, dPS-Y2 and dPS-Y3) and green (cultivar Gloriosa, dPS-G1, dPS-G2 and dPS-G3) seed embryos after depletion of highly-abundant proteins with 0.07% (w/v) protamine sulphate. Reference samples of undigested proteins (5 µg), isolated from yellow and green seeds were loaded on lines 2 (PS-Y) and 6 (PS-G), respectively; St, mixture of molecular weight standards

**
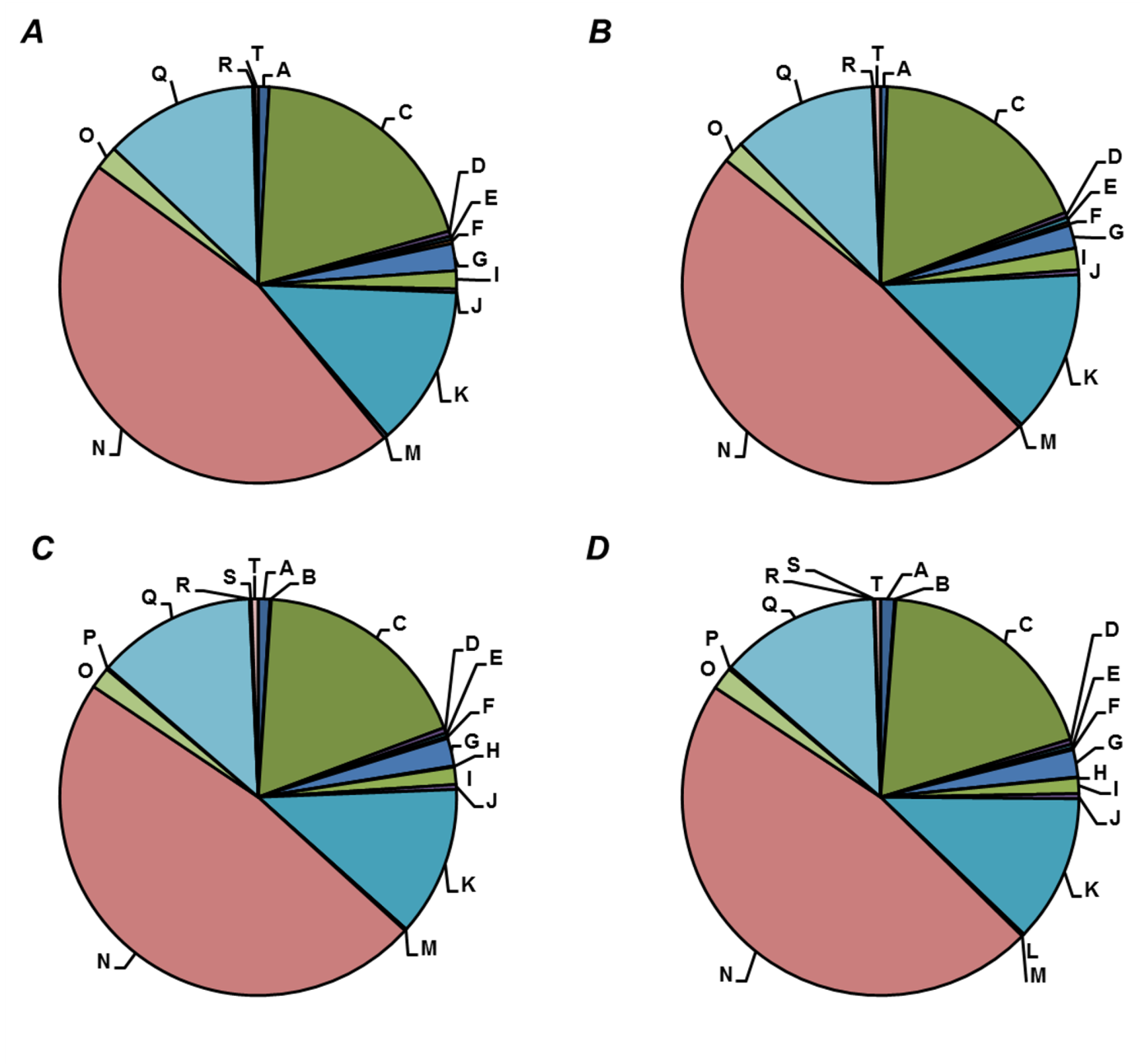
**

Figure S1-8 Prediction of sub-cellular localization of proteins, identified in the embryos of yellow seeds (cultivar Millennium, B and D) and in those, isolated from green seeds (cultivar Gloriosa, A and C) in non-depleted samples (C,D) and after treatment with 0.07% (w/v) protamine sulfate (A,B). The letters denote sub-cellular localization: A, cytoskeleton; B, cytoskeleton and cytoplasm; C, cytoplasm; D, cytoplasm and mitochondria; E, cytoplasm and nuclear; F, cytoplasm and plasma membrane; G, endoplasmic reticulum; H, endoplasmic reticulum and Golgi apparatus; I, extracellular/secreted; J, Golgi apparatus; K, mitochondria; L, mitochondria and nuclear; M, mitochondria and cytoplasm; N, nuclear; O, nuclear and cytoplasm; P, nuclear and plasma membrane; Q, plasma membrane; R, plasma membrane and cytoplasm; S, plasma membrane and endoplasmic reticulum; T, peroxisome.

# Literature

1. Lichtenthaler, H. K.; Wellburn, A. R. Determinations of total carotenoids and chlorophylls a and b of leaf extracts in different solvents. *Biochemical Society Transactions* **1983**, *11*, 591–592, doi:10.1042/bst0110591.

2. Bilova, T.; Paudel, G.; Shilyaev, N.; Schmidt, R.; Brauch, D.; Tarakhovskaya, E.; Milrud, S.; Smolikova, G.; Tissier, A.; Vogt, T.; Sinz, A.; Brandt, W.; Birkemeyer, C.; Wessjohann, L. A.; Frolov, A. Global proteomic analysis of advanced glycation end products in the Arabidopsis proteome provides evidence for age-related glycation hot spots. *J. Biol. Chem.* **2017**, *292*, 15758–15776, doi:10.1074/jbc.M117.794537.

3. Frolov, A.; Bilova, T.; Paudel, G.; Berger, R.; Balcke, G. U.; Birkemeyer, C.; Wessjohann, L. A. Early responses of mature Arabidopsis thaliana plants to reduced water potential in the agar-based polyethylene glycol infusion drought model. *J. Plant Physiol.* **2017**, *208*, 70–83, doi:10.1016/j.jplph.2016.09.013.

4. Spiller, S.; Frolov, A.; Hoffmann, R. Quantification of Specific Glycation Sites in Human Serum Albumin as Prospective Type 2 Diabetes Mellitus Biomarkers. *Protein Pept. Lett.* **2017**, *24*, 887–896, doi:10.2174/0929866524666170202124120.

5. Szklarczyk, D.; Franceschini, A.; Wyder, S.; Forslund, K.; Heller, D.; Huerta-Cepas, J.; Simonovic, M.; Roth, A.; Santos, A.; Tsafou, K. P.; Kuhn, M.; Bork, P.; Jensen, L. J.; von Mering, C. STRING v10: protein-protein interaction networks, integrated over the tree of life. *Nucleic Acids Res.* **2015**, *43*, D447-452, doi:10.1093/nar/gku1003.

6. Krishnakumar, V.; Hanlon, M. R.; Contrino, S.; Ferlanti, E. S.; Karamycheva, S.; Kim, M.; Rosen, B. D.; Cheng, C.-Y.; Moreira, W.; Mock, S. A.; Stubbs, J.; Sullivan, J. M.; Krampis, K.; Miller, J. R.; Micklem, G.; Vaughn, M.; Town, C. D. Araport: the Arabidopsis Information Portal. *Nucleic Acids Res* **2015**, *43*, D1003–D1009, doi:10.1093/nar/gku1200.

7. Kim, Y. J.; Wang, Y.; Gupta, R.; Kim, S. W.; Min, C. W.; Kim, Y. C.; Park, K. H.; Agrawal, G. K.; Rakwal, R.; Choung, M.-G.; Kang, K. Y.; Kim, S. T. Protamine sulfate precipitation method depletes abundant plant seed-storage proteins: A case study on legume plants. *Proteomics* **2015**, *15*, 1760–1764, doi:10.1002/pmic.201400488.
